# Origin and evolution of *MIR1444* genes in Salicaceae

**DOI:** 10.1038/srep39740

**Published:** 2017-01-10

**Authors:** Meizhen Wang, Caili Li, Shanfa Lu

**Affiliations:** 1 Institute of Medicinal Plant Development, Chinese Academy of Medical Sciences and Peking Union Medical College, Beijing, China

## Abstract

miR1444s are functionally significant miRNAs targeting polyphenol oxidase (*PPO*) genes for cleavage. *MIR1444* genes were reported only in *Populus trichocarpa*. Through the computational analysis of 215 RNA-seq data, four whole genome sequences of Salicaceae species and deep sequencing of six *P. trichocarpa* small RNA libraries, we investigated the origin and evolution history of *MIR1444s*. A total of 23 *MIR1444s* were identified. *Populus* and *Idesia* species contain two *MIR1444* genes, while *Salix* includes only one. *Populus* and *Idesia MIR1444b* genes and *Salix MIR1444s* were phylogenetically separated from *Populus* and *Idesia MIR1444a* genes. Ptr-miR1444a and ptr-miR1444b showed sequence divergence. Compared with ptr-miR1444b, ptr-miR1444a started 2 nt upstream of precursor, resulting in differential regulation of *PPO* targets. Sequence alignments showed that *MIR1444* genes exhibited extensive similarity to their *PPO* targets, the characteristics of *MIRs* originated from targets through an inverted gene duplication event. Genome sequence comparison showed that *MIR1444* genes in *Populus* and *Idesia* were expanded through the Salicoid genome duplication event. A copy of *MIR1444* gene was lost in *Salix* through DNA segment deletion during chromosome rearrangements. The results provide significant information for the origin of plant miRNAs and the mechanism of Salicaceae gene evolution and divergence.

*MIR1444* genes were first reported in 2008 in *Populus trichocarpa*^1^, a model tree species with the whole genome sequence available^2^. Like other microRNA genes (*MIRs*), after transcription from the *MIR1444* loci by RNA polymerase II, long *MIR1444* transcripts are processed into 21-nucleotide mature microRNAs (miRNAs), termed miR1444s, under the catalysis of Dicer-like 1 (DCL1) in interaction with several other proteins^3^,^4^,^5^,^6^. Mature miR1444s target a subset of polyphenol oxidase genes (*PPOs*) for cleavage in *Populus trichocarpa*, playing significant regulatory roles in copper homeostasis and stress responses in plants^1^,^7^,^8^,^9^.

Compared with those deeply conserved old miRNAs, such as miR156, miR159, miR171, miR390 and miR408 inherited from the ancestral embryophyte and miR162, miR164, miR397, miR398, miR399 and miR482 evolved in the ancestral spermatophyte^10^, miR1444s are evolutionarily young. *MIR1444* genes were reported only in *P. trichocarpa*^1^,^11^. Mature miR1444 sequences were isolated only from *Salix matsudana*^12^ and various *Populus* species, such as *P. trichocarpa*^1^,^11^, *P. euphratica*^13^, *P. tomentosa*^14^,^15^, *P. beijingensis*^16^, and *P. szechuanica*^17^. Both *Populus* and *Salix* are members of the family Salicaceae.

Salicaceae is a family of dioecious flowering plants. Classically, it includes two genera, *Populus* and *Salix*, while recent molecular data has shown a close relationship among *Populus* and *Salix* and many genera formerly placed in Flacourtiaceae, such as *Idesia, Dovyalis*, and *Azara*^18^,^19^,^20^. The family Flacourtiaceae is now dismembered, and many of its members are placed in Salicaceae, expanding the circumscription of the family Salicaceae to contain about 1000 species in 55 genera^21^. All of them are woody trees or shrubs.

Due to high economic and ecological significance of Salicaceae species, great efforts have been made to decode their genomes and transcriptomes. So far, the whole genomes of four Salicaceae species, including two poplars (*P. trichocarpa* and *P. euphratica*) and two willows (*S. purpurea* and *S. suchowensis*), have been sequenced^2^,^22^,^23^. Comparative sequence analysis of genomes demonstrated that two ancient whole-genome duplication (WGD) events occurred in *Populus* and *Salix*. The more recent WGD, known as the Salicoid duplication event, took place around 60 to 65 million years ago (Ma), affecting roughly 92% of the *P. trichocarpa* genome^2^. The divergence between *Populus* and *Salix* happened around 45 to 52 Ma^23^,^24^,^25^, while the divergence between *P. trichocarpa* and *P. euphratica* occurred about 14 Ma^22^. In addition to the genomes, huge transcriptome data has been generated for *Populus, Salix* and *Idesia*. It provides a foundation for genome- and transcriptome-wide analysis of gene evolution.

With the aim to elucidate the possible origin and evolution history of *MIR1444* genes in Salicaceae, we investigated *MIR1444* genes and their *PPO* targets in three phylogenetically related genera, including *Populus, Salix* and *Idesia*. Based on the results from integrative analysis of the whole genome sequences of *P. trhichcarpa, P. euphratica, S. purpurea* and *S. suchowensis*, transcriptome data from 215 high-throughput 454 and illumina RNA-seq libraries and sequence data from molecular cloning, we conclude that *Populus* and *Idesia* contain two *MIR1444* genes, while *Salix* includes only one. Plant *MIR1444* genes were originated from their target *PPO* genes through an inverted gene duplication event. *MIR1444* genes in *Populus* and *Idesia* were expanded through the Salicoid genome duplication event and diverged during the evolution of Salicaceae plants. The lost of a *MIR1444* gene copy in *Salix* was resulted from DNA segment deletion after the divergence of *Populus* and *Salix* lineages.

## Results

### Genome- and transcriptome-wide identification of *MIR1444* genes in *Populus*

A total of five *MIR1444* genes, termed *ptr*-*MIR1444a* – *ptr*-*MIR1444e*, were predicted previously in *P. trichocarpa*^1^,^11^,^26^. In order to test whether all of the five *ptr*-*MIR1444* genes are authentic, we performed blast analysis of *ptr*-*MIR1444* precursors against the *P. trichocarpa* genome database (v3.0, https://phytozome.jgi.doe.gov/pz/portal.html)^2^. The results mapped *ptr*-*MIR1444a* to chromosome 8 and *ptr*-*MIR1444b*–*ptr*-*MIR1444e* to a genomic locus on chromosome 10 (Fig. S1). *Ptr*-*MIR1444c* shows 100% identities with the assembled genome sequence, while *ptr*-*MIR1444b* has one nucleotide mismatch located in the loop region of fold-back structure. The mature ptr-miR1444b and ptr-miR1444c sequences are identical. *Ptr*-*MIR1444d* is an anti-sense sequence of *ptr*-*MIR1444c*, while *ptr*-*MIR1444e* is an anti-sense sequence of *ptr*-*MIR1444b*. Further, we searched the Nt and EST databases (http://blast.ncbi.nlm.nih.gov/Blast.cgi) and 318.6 million of RNA-seq reads from five illumina runs for *ptr*-*MIR1444a*–*ptr*-*MIR1444c* (Table S1)^27^,^28^. Expressed *P. trichocarpa* sequences identical to *ptr*-*MIR1444a* and *ptr*-*MIR1444b* were found. No sequence identical to *ptr*-*MIR1444c* was retrieved, although *ptr*-*MIR1444c* exhibited 100% identities with the assembled *P. trichocarpa* genome sequence. Taken together, we conclude that the *P. trichocarpa* genome contains two *ptr*-*MIR1444* genes, *ptr*-*MIR1444a* in chromosome 8 and *ptr*-*MIR1444b* in chromosome 10. There is a mis-assembled nucleotide in the *ptr*-*MIR1444b* locus in *P. trichocarpa* genome v3.0.

**Figure 1:**
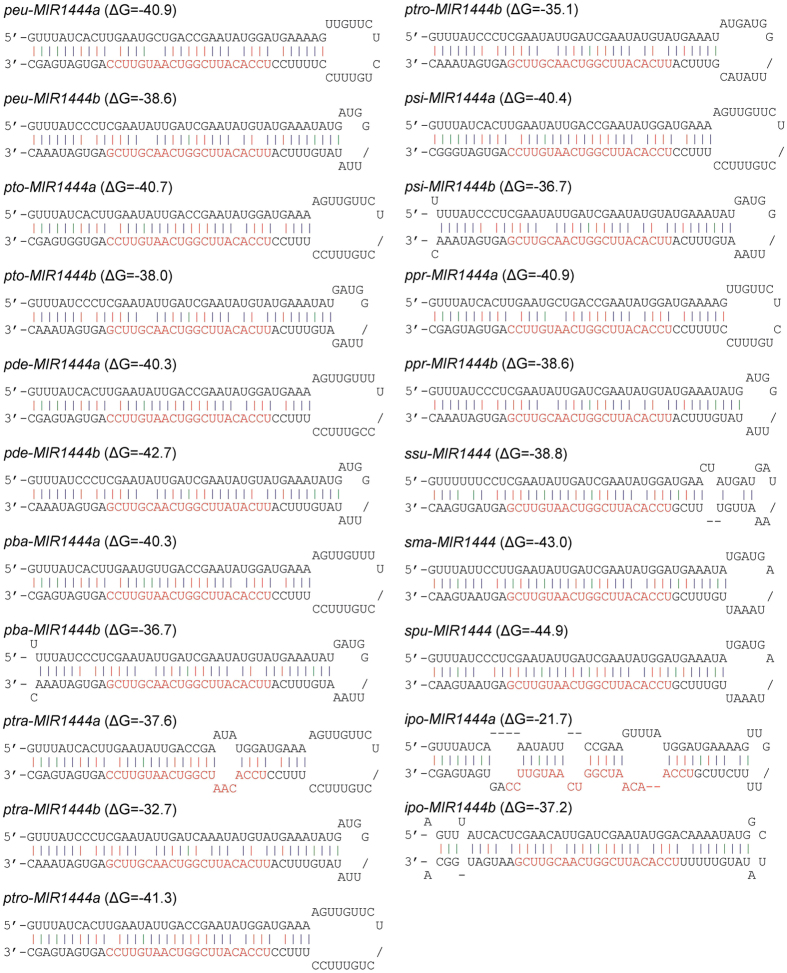
Hairpin structures of *MIR1444* precursors in *Populus, Salix* and *Idesia*. Mature miRNA sequences are shown in red. *Peu: P. euphratica, pto: P. tomentosa, pde: P. deltoids, pba: P. balsamifera, ptra: P. tremula, ptro: P. tremuloides, psi: P. simonii, ppr: P. pruinosa, ssu: S. suchowensis, sma: S. matsudana, spu: S. purpurea, ipo: I. polycarpa.*

*P. euphratica*, native to desert regions ranging from western China to North Africa, is another *Populus* species with the whole genome sequencs^22^. We performed blast analysis of *P. trichocarpa MIR1444s* against the *P. euphratica* genome database^29^ (Populus euphratica_1.1, http://me.lzu.edu.cn/stpd/). Two *P. euphratica peu*-*MIR1444* genes were identified (Fig. 1). We then searched for *peu*-*MIR1444a* and *peu*-*MIR1444b* in the Nt and EST databases (http://blast.ncbi.nlm.nih.gov/Blast.cgi), 769.8 million of RNA-seq reads from 24 illumina runs and 505.7 thousand of sequence reads from two 454 runs (Table S1)^30^,^31^,^32^,^33^,^34^,^35^. *peu*-*MIR1444a* and *peu*-*MIR1444b* precursors were successfully identified from transcriptomes, confirming the existence of two expressed *MIR1444s* in *P. euphratica*.

Using similar strategies, we identified two expressed *MIR1444* genes from each of the other *Populus* species without whole genome sequences, including *P. tomentosa, P. deltoids, P. balsamifera, P. tremula, P. tremuloides, P. simonii*, and *P. pruinosa* (Fig. 1; Table S1). It is consistent with the results from *P. trichocarpa* and *P. euphratica*, suggesting the existence of two *MIR1444* genes in a genome of *Populus* species.

### Only one *MIR1444* gene in *S. Purpurea, S. Suchowensis* and *S. Matsudana*

*Salix*, comprising more than 300 species, is the genus phylogenetically most closely related to *Populus*^36^. Based on the fossil record and comparative genomic analysis, the two sister genera of Salicaceae were estimated to diverge from each other approximately 45 to 52 Ma^23^,^24^,^25^. The whole genomes of two *Salix* species, including *S. purpurea* native to Europe and western Asia and *S. suchowensis* native to the north of China, have been sequenced recently^23^ (https://phytozome.jgi.doe.gov/pz/portal.html). Since no *Salix MIR1444* gene had been reported previously, we first searched current assemblies of the *S. purpurea* and the *S. suchowensis* genomes. A *MIR1444* gene was identified from each of the two *Salix* genomes (Fig. 1). We next carried out blast analysis of the identified *spu-* and *ssu-MIR1444* sequences against the high-throughput illumina and 454 sequencing data of *S. purpurea* and *S. suchowensis* (Table S1). The results showed that *spu-MIR1444* and *ssu-MIR1444* were the expressed *MIR1444* genes in *S. purpurea* and *S. suchowensis*, respectively.

*S. matsudana*, also known as Chinese willow, is a species of willow native to northwest of China. Although its genome has not been sequenced, there are 228.7 million of RNA-seq reads from five illumina runs available (Table S1)^12^,^37^. Blast analysis of *MIR1444* sequences against RNA-seq data of *S. matsudana* showed the existence of an expressed *MIR1444* gene, termed *sma-MIR1444*, in *S. matsudana* (Fig. 1). Taken together, the results suggest that there is only one *MIR1444* gene in a genome of *Salix* species, such as *S. suchowensis, S. purpurea*, and *S. matsudana*.

### Molecular cloning of *Idesia Polycarpa MIR1444* precursors and phylogenetic analysis of Salicaceae *MIR1444s*

*Idesia* is a genus formerly placed in the family Flacourtiaceae, but now included in the family Salicaceae^21^. *Idesia* comprises the single species *I. polycarpa* Maximowicz, which is native to eastern Asia, including China, Japan and Korea. Molecular and morphological evidence has shown that *Idesia* is the most closely related taxa to *Populus* and *Salix*^18^,^19^,^21^. Although the genome of *I. polycarpa* has not been decoded, transcriptome of male and female flower buds has been sequenced using high-throughput Illumina HiSeq 2000 (SRX1421098). Blast analysis of *Populus* and *Salix MIR1444* genes against RNA-seq data of *I. polycarpa* flower buds identified partial sequences of two *ipo-MIR1444* genes. We then designed primers based on the partial sequences and carried out PCR of *ipo-MIR1444* precursors. The results suggest that *I. polycarpa* contains two *MIR1444* genes, termed *ipo-MIR1444a* and *ipo-MIR1444b*, respectively (Fig. 1).

**Figure 2:**
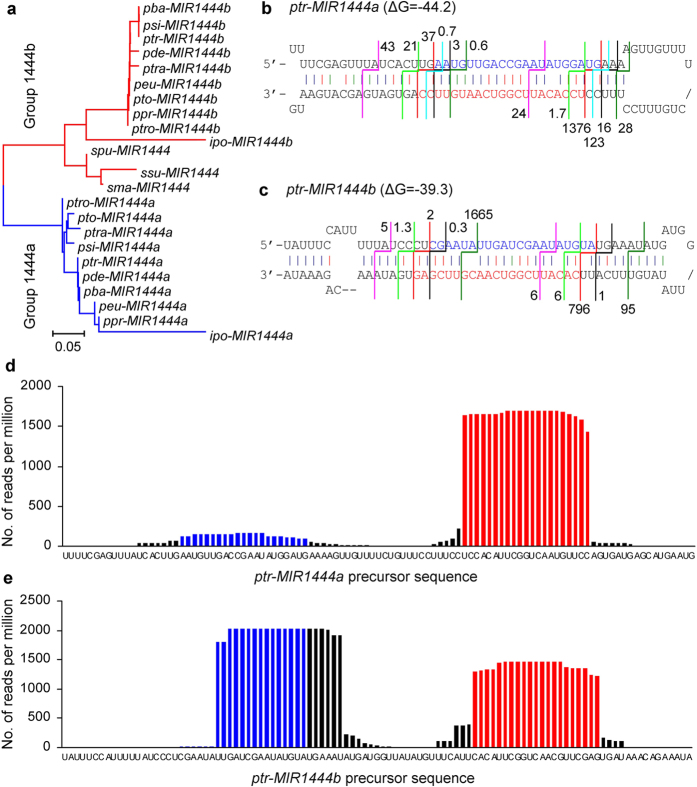
Conservation and divergence of *MIR1444s*. (**a**) Phylogenetic relationship of *MIR1444* precursors in various *Populus, Salix* and *Idesia* species. It includes *P. euphratica (peu*), *P. tomentosa (pto*), *P. deltoids (pde*), *P. balsamifera (pba*), *P. tremula (ptra*), *P. tremuloides (ptro*), *P. simonii (psi*), *P. pruinosa (ppr*), *S. suchowensis (ssu*), *S. matsudana (sma*), *S. purpurea (spu*), and *I. polycarpa (ipo*). Groups 1444a and 1444b indicate two groups identified. (**b**) and (**c**) Hairpin structures of *ptr-MIR1444a* (**b**) and *ptr-MIR1444b* (**c**). miR1444a and miR1444b are shown in red. miR1444a* and miR1444b* are shown in blue. The 5′ and 3′ ends of miRNA/miRNA* pairs identified in the small RNA libraries are shown by broken lines with same color. The number of reads per million is shown at the 5′ end of each 21 nt small RNA identified. (**d**) and (**e**) High throughput sequencing analysis of small RNAs from *ptr-MIR1444a* (**d**) and *ptr-MIR1444b* (**e**) precursors.

Through genome- and transcriptome-wide analysis and molecular cloning, we identified a total of 23 *MIR1444* genes in *Populus, Salix* and *Idesia* (Fig. 1). A neighbor-joining (NJ) phylogenetic tree for precursor sequences of the identified *MIR1444s* was constructed using MEGA5.0^38^ (Fig. 2a). *MIR1444s* could be divided into two groups, 1444a and 1444b. *Salix MIR1444s* cluster with *Populus* and *Idesia MIR1444b* precursors in the 1444b group, while *Populus* and *Idesia MIR1444a* precursors cluster together in the 1444a group (Fig. 2a). It suggests that *Salix MIR1444s* are homologs of *Populus* and *Idesia MIR1444b* genes. The homolog of *Populus* and *Idesia MIR1444a* genes was lost in *Salix* plants.

### Divergence of mature miR1444a and miR1444b in *P. trichocarpa*

Comparison of *ptr-MIR1444a* and *ptr-MIR1444b* precursors showed sequence divergence, which led to varied stem-loop structures with different stabilities (ΔG) (Fig. 2b,c). ptr-miR1444s and their corresponding miRNA* sequences are relatively conserved, while the nucleotides outside of this region show greater variation. Comparison of ptr-miR1444a and ptr-miR1444b showed two nucleotide changes (U-to-C, C-to-G) near the 3′ end. Variation at three locations were found between ptr-miR1444a* and ptr-miR1444b*.

In order to further analyze the mature miR1444s in *P. trichocarpa*, we sequenced six small RNA libraries using high-throughput Illumina sequencing technology. A total of 323,577,100 raw reads were obtained. After removing adaptors and low quality sequences, 211,425,411 clean reads represented by 30,938,856 unique small RNA sequences were obtained. We mapped the unique small RNA sequences to the precursors of ptr-miR1444a and ptr-miR1444b using SOAP2 with no mismatch allowed, respectively^39^. Of the 244 small RNA hits on ptr-miR1444a precursor, six different miRNA/miRNA* duplexes were found (Fig. 2b). Among them, ptr-miR1444a showed an extremely high abundance of 1,376 RPM (reads per million), accounting for 74.08% of the reads mapping to that site. Similarly, five distinct miRNA/miRNA* pairs were obtained from the 171 small RNA hits on ptr-miR1444b precursor (Fig. 2c). Ptr-miR1444b had 796 RPM, accounting for 25.38% of the mapped reads. Compared to ptr-miR1444a, the expression of ptr-miR1444b is lower in plantlets. Interestingly, we found that ptr-miR1444a started 2 nt upstream relative to ptr-miR1444b (Fig. 2b,c). Details on the location of ptr-miR1444a/b and their corresponding miRNAs* are shown in Fig. 2d and e, respectively. Sequence divergence between ptr-miR1444a and ptr-miR1444b was confirmed by mapping sequence reads from three previously reported small RNA libraries^40^ (GSM717875, GSM717876 and GSM717877) to *ptr-MIR1444* precursors (Fig. S2). It should be noticed that, in addition to ptr-miR1444b, the *ptr-MIR1444b* precursor generates another 21 nt small RNA with high sequence reads (1,665 RPM) in our small RNA libraries (Fig. 2c). This small RNA was produced from the 5′ arm of ptr-MIR1444b. The function of this small RNA remains to be elucidated. Taken together, the results suggest that the natural variation between *ptr-MIR1444a* and *ptr-MIR1444b* may affect foldback structures and miRNA biogenesis.

### Identification and characterization of *PPO* genes in *Populus* and *Salix*

It has been shown that mature miR1444s target *PPO* genes for cleavage in *P. trichocarpa*^1^,^7^,^8^. PPOs are copper-binding enzymes catalyzing the dehydrogenation of catechols to the corresponding *o*-quinones^41^. It also act as bifunctional enzymes to oxidize monophenols first to *o*-diphenol intermediates and then to the corresponding *o*-quinones^41^. Plant PPO proteins typically consist of N-terminal targeting signal, dicopper centre and C-terminal region^42^,^43^. The dicopper centre contains two copper-binding domains, CuA and CuB, each of which is approximately 50 amino acids in length. Analysis of the *PPO* gene families in 25 sequenced genomes from chlorophytes, bryophytes and lycophytes showed that the number of *PPO* genes in a genome varied among 0 to 13^43^. No *PPO* genes were found in *Arabidopsis*. In order to identify miR1444-targeted *PPO* genes, we searched current assemblies of the *P. trichocarpa* (v3.0), *P. euphratica* (v1.0), *S. purpurea* (v1.0) and *S. suchowensis* (v1.0) genomes^2^,^22^,^23^ (https://phytozome.jgi.doe.gov/pz/portal.html). We identified a total of 34 full-length *PPO* genes, including 15 from *P. trichocarpa*, 6 from *P. euphratica*, 9 from *S. purpurea* and 4 from *S. suchowensis* (Table 1). Compared with *P. trichocarpa* and *S. purpurea*, the number of full-length *PPO* genes in *P. euphratica* and *S. suchowensis* were significantly less. It could be due to the incompleteness of the current assemblies of the *P. euphratica* and *S. suchowensis* genomes. In addition to the full-length genes, a total of 26 partial *PPO* sequences were identified in the *P. trichocarpa, P. euphratica, S. purpurea* and *S. suchowensis* genomes (Table S2). Some of the identified partial sequences could result from the incompleteness of genome assemblies, while a significant proportion appeared to be pseudogenes.

**Figure 3:**
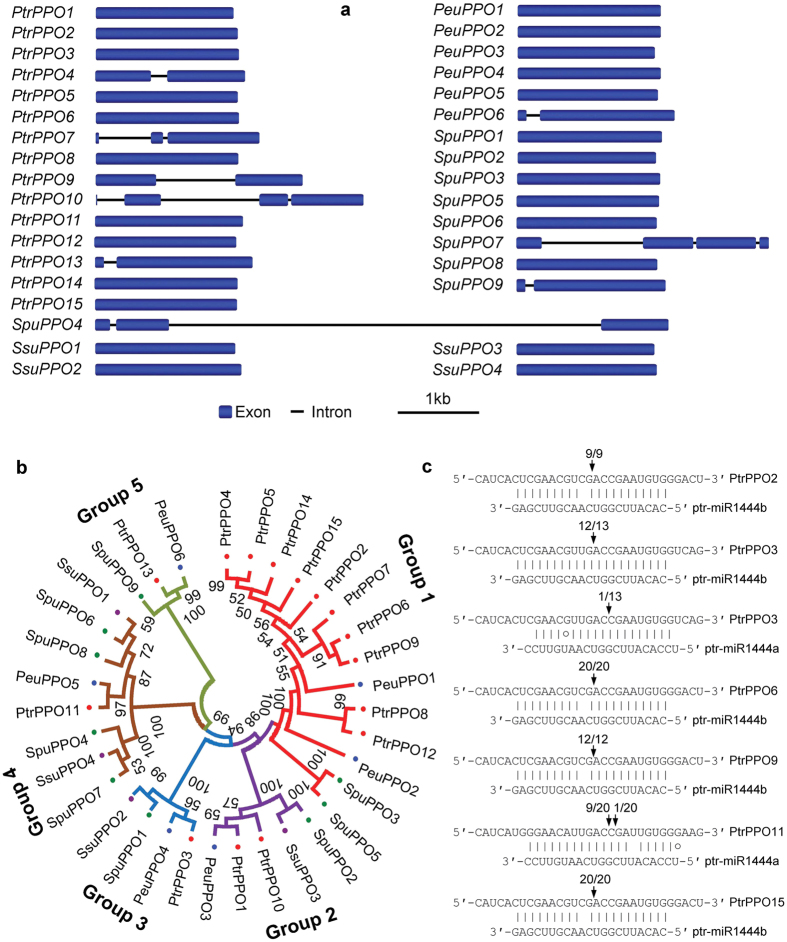
miR1444-mediated cleavage of *PPOs* in *Populus* and *Salix*. (**a**) Gene structures of *PPOs*. (**b**) Unrooted neighbor-joining tree of PPOs. Five groups identified are shown. (**c**) Validation of miR1444-mediated cleavage of *PPOs* in *P. trichocarpa*. The mRNA cleavage sites were determined by the modified 5′ RLM-RACE method. The mRNA sequence of each complementary site from 5′ to 3′ and the ptr-miR1444a and ptr-miR1444b sequences from 3′ to 5′ are shown. Watson-Crick pairing (vertical dashes) and G:U wobble pairing (circles) are indicated. Vertical arrows indicate the 5′ termini of miRNA-guided cleavage products with the frequency of clones shown.

Among the 34 *PPO* genes, *PtrPPO10* and *SpuPPO7* contain three introns, *PtrPPO7* and *SpuPPO4* have two introns, *PtrPPO4, PtrPPO9, PtrPPO13, PeuPPO6* and *SpuPPO9* include an intron, while the other 25 *PPOs* are single exon genes (Fig. 3a). We constructed a neighbor-joining (NJ) phylogenetic tree for the deduced PPO proteins using MEGA5.0^38^ (Fig. 3b). As a result, the identified 34 *Populus* and *Salix* PPOs could be divided into 5 groups. Group 1 is the largest one. It contains 14 PPOs, including 10 from *P. trichocarpa*, two from *P. euphratica*, and two from *S. purpurea* (Fig. 3b). Groups 2 and 3 contain five and four PPOs, respectively. These PPOs are from four different plant species. Group 4 contain eight PPOs, four of which are from *S. purpurea*. The three PPOs containing a secretory pathway signal peptide, including PtrPPO13, PeuPPO6 and SpuPPO9, are clustered in group 5 (Fig. 3b, Table 1).

### miR1444-mediated cleavage of *PPO* transcripts

**Figure 4:**
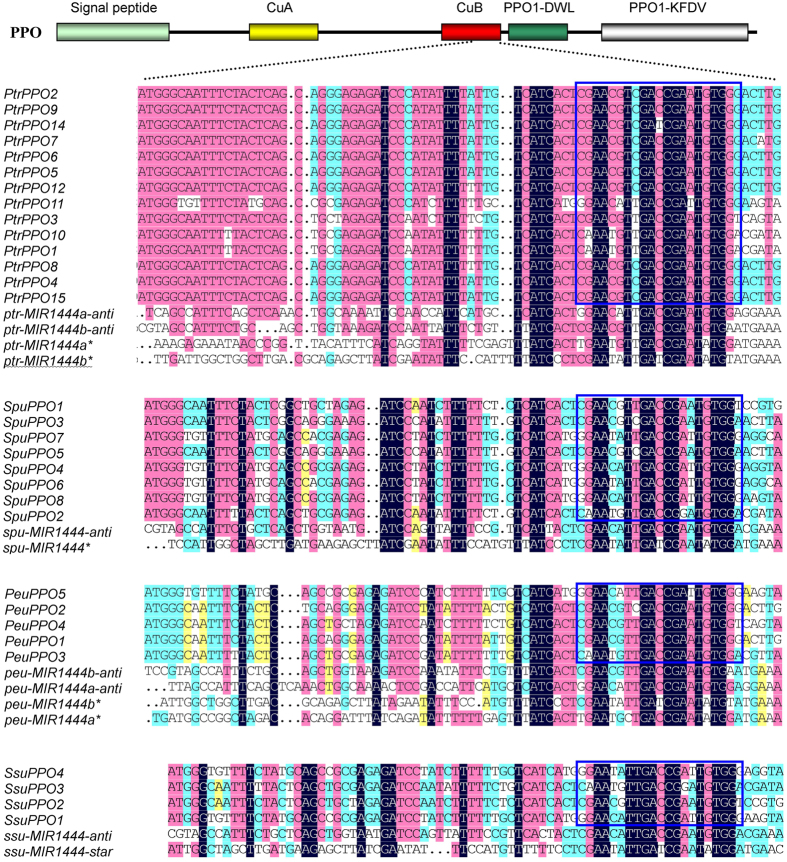
Multiple sequence alignment of *MIR1444* precursors and miR1444-targeted *PPOs* in *Populus* and *Salix*. The schematic diagram of *PPOs* is shown. cDNA regions coding for conserved domains are shown and indicated by heavy lines with different colors. Mature miRNAs of the *MIR1444* gene family target to the cDNA region encoding CuB domain. Sequence alignments of the sense orientation of *PPOs* and the miRNA* foldback arms and the antisense sequences of miRNA foldback arms are shown in the expanded region. Blue boxes indicate miRNA complementary sites.

Plant miRNAs have perfect or near-perfect complementarities to their targets, allowing an effective prediction of target sequence through computational approaches like psRNATarget^44^,^45^. Among the 34 identified *PPO* genes, complementary sequences of miR1444s were found in 31 genes (Fig. 4). It indicates that 31 of the 34 *PPO* genes are potential targets of miR1444s. The target sites locate in regions encoding CuB, a conserved domain of PPO proteins (Fig. 4). Analysis of the subcellular localization of PPO proteins using TargetP 1.1^46^ showed that the majority of the predicted targets of miR1444 contained a chloroplast transit peptide at the N-terminus, while the three non-targets of miR1444s included a secretory pathway signal peptide (Table 1).

Plant miRNAs regulate target gene expression mainly through direct cleavage of target mRNAs at the 10^th^ miRNA nucleotide from the 5′ end^44^,^47^. To validate miRNA-mediated cleavage of predicted targets, we carried out rapid amplification of 5′ complementary DNA ends (5′-RACE) for fourteen *PPO* genes on mRNAs isolated from leaves and xylem of *P. trichocarpa* as described previously^48^. Six of the *PPO* genes tested were indeed cleaved by miR1444s *in vivo* (Fig. 3c), verifying the results from computational prediction (Fig. 4). Examination of the cleavage sites showed that *PtrPPO11* was cleaved by ptr-miR1444a, *PtrPPO2, PtrPPO6, PtrPPO9* and *PtrPPO15* were regulated by ptr-miR1444b, while *PtrPPO3* was targeted by both ptr-miR1444a and ptr-miR1444b. It should be noted that the frequency of ptr-miR1444a-guided cleavage products of *PtrPPO3* (12/13) is much higher than that of ptr-miR1444b (1/13). Similar results have been obtained previously^1^. It indicates that *PtrPPO3* transcripts are predominantly cleaved by ptr-miR1444a in the plant tissues analyzed, although they are also targets of ptr-miR1444b. Taken together, the results confirm the divergence of mature ptr-miR1444a and ptr-miR1444b, and suggest that both of them are functional.

### *MIR1444* genes show extensive similarity to *PPO* targets

In *Arabidopsis*, several *MIRs*, such as *MIR161* and *MIR163*, were generated from inverted gene duplication events of target genes^49^,^50^. To elucidate the origin of *MIR1444* genes, we aligned foldback arms and their flanking sequences of *MIR1444* precursors with *PPO* targets. Similarity was detected among miRNA*-containing 5′ arms, complementary sequences of miRNA-containing 3′ arm, and 78 nt *PPO* sequences containing target sites and partial of CuB conserved domain-encoding sequences (Fig. 4). The complementarity or similarity to *PPO* targets was relatively high in the miRNA and miRNA* regions (Fig. 4), suggesting they were under evolutionary constraints and indicating the significance of miR1444s. Compared with miRNA*-containing 5′ arms, complementary sequences of miRNA-containing 3′ arms showed higher similarity to *PPO* targets. *P. trichocarpa ptr-MIR1444a* and *ptr-MIR1444b* showed the greatest similarity (~70%) with the three *PPO* genes located in chromosome 11, including *PtrPPO1, PtrPPO3* and *PtrPPO10* (Fig. 4). *spu-MIR1444* showed the greatest similarity with *SpuPPO1* and *SpuPPO2* in *S. purpurea. ssu-MIR1444* showed the greatest similarity with *SsuPPO2* and *SsuPPO3* in *S. suchowensis*. All of the *PPO* genes with the greatest similarity with the corresponding *MIR1444s* are members of groups 2 and 3 (Fig. 3b). It indicates that *MIR1444s* and *PPO* genes in these groups probably have a common ancestor. Among the four *P. trichocarpa* and *P. euphratica MIR1444* genes, *MIR1444b* showed greater similarity with *PPO* genes than *MIR1444a* in *P. trichocarpa* and *P. euphratica*, respectively. It indicates that *MIR1444b* is more conserved than *MIR1444a*.

### Expansion of *MIR1444* genes through the salicoid genome duplication event

**Figure 5:**
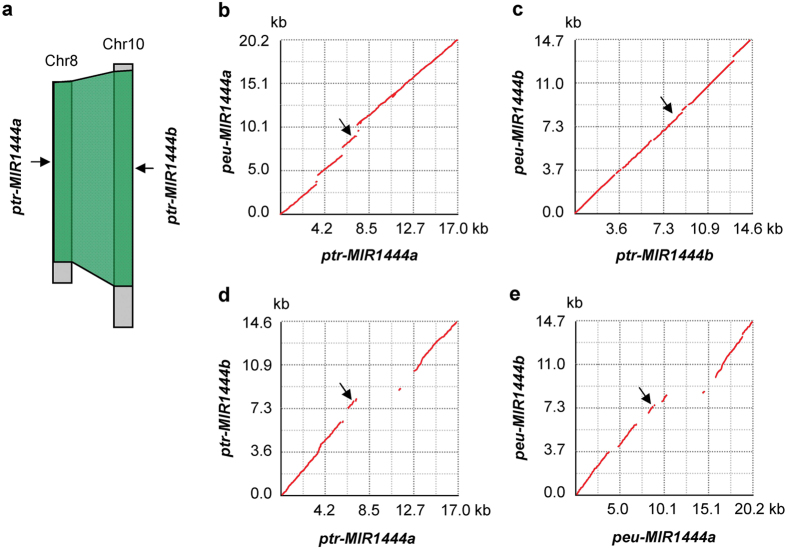
Duplication and divergence of *MIR1444* genes in *Populus*. (**a**) Locations of *P. trichocarpa (ptr) MIR1444a* in chromosome 8 (chr8) and *MIR1444b* in chr10. The schematic diagram of two homologous genome blocks (green) arising from the Salicoid duplication event was adapted from Tuskan *et al*.^9^. (**b**–**e**) Dot-plots of genomic DNA segments in *P. trichocarpa* and *P. euphratica (peu*). The alignment and visualization of two *MIR1444s* and/or surrounding genomic DNA sequences was performed using zPicture (http://zpicture.dcode.org/). Arrows indicate the corresponding locations of miR1444a and miR1444b.

Analysis of the fourfold synonymous third-codon transversion position (4DTV) values in *P. trichocarpa* and *P. euphratica* has shown that two ancient whole-genome duplication (WGD) events occurred in the *Populus* lineage^2^,^22^. The recent WGD event, known as the Salicoid duplication event, was estimated to occur around 60 to 65 Ma, and both genera *Salix* and *Populus* shared this event^2^,^22^,^23^. *P. trichocarpa MIR1444a* in chromosome 8 and *MIR1444b* in chromosome 10 are located in two homologous genome blocks arising from the Salicoid duplication event^2^ (Fig. 5a). Sequence alignments using zPicture (http://zpicture.dcode.org/) and Generic Synteny Browser (GBrowse-syn) (http://me.lzu.edu.cn/stpd/#main_tabs=2) showed that the genomic DNA segments of *peu-MIR1444a* in scaffold 1.1 and *peu-MIR1444b* in scaffold 9.1 shared high level of sequence homology with the segments of *ptr-MIR1444a* in chromosome 8 and *ptr-MIR1444b* in chromosome 10, respectively (Fig. 5b,c). The results indicate the expansion of *MIR1444* genes through the Salicoid duplication event.

Although *MIR1444a* and *MIR1444b* are located in genome blocks arising from the Salicoid duplication event in *P. trichocarpa* and *P. euphratica*, significant divergence has occurred in the promoter and downstream regions of *MIR1444a* and *MIR1444b* genes (Fig. 5d,e). In contrast, the homologies are higher between genomic DNA regions surrounding *ptr-MIR1444a* and *peu-MIR1444a* and between the regions surrounding *ptr-MIR1444b* and *peu-MIR1444b* (Fig. 5b,c). The difference of sequence similarities is consistent with the difference of estimated time of the Salicoid duplication event (around 60 to 65 Ma) and *P. trichocarpa* and *P. euphratica* divergence (about 14 Ma)^2^,^22^,^23^.

### Loss of *MIR1444a* homologs in *Salix* through DNA segment deletion

**Figure 6:**
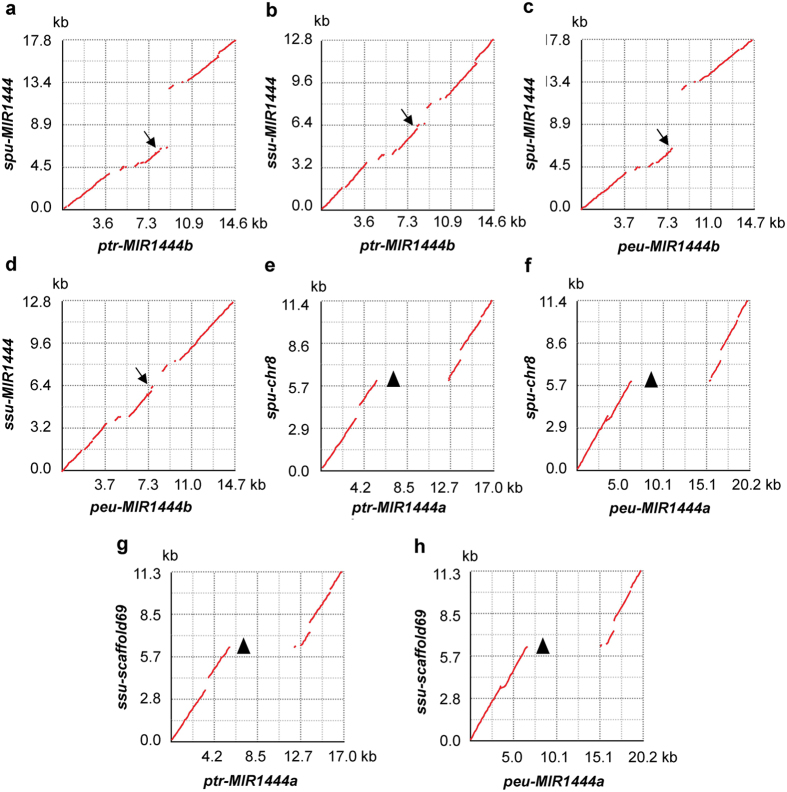
Dot-plots of genomic DNA segments in *Salix* and *Populus*. The alignment and visualization of two *MIR1444s* and/or surrounding genomic DNA sequences was performed using zPicture (http://zpicture.dcode.org/). Arrows indicate the corresponding locations of mature miR1444s in *Salix* and miR1444bs in *Populus*. Triangles show the positions of *P. trichocarpa (ptr*) and *P. euphratica (peu*) miR1444as that are absent from *S. purpurea (spu*) chromosome 8 (chr8) and *S. suchowensis (ssu*) assembled genomic DNA scaffold69.

Although the Salicoid duplication event took place before the divergence of the *Populus* and *Salix* lineages, only one *MIR144* gene was identified in the genomes of *S. purpurea* and *S. suchowensis* (Fig. 1). Sequence comparison analysis showed that the genomic regions of *MIR1444s* in *S. purpurea* chromosome 10 and *S. suchowensis* scaffold 90 were highly similar to *MIR1444b* in *P. trichocarpa* chromosome 10 and *P. euphratica* scaffold 9.1 (Fig. 6a–d). It suggests that *Salix MIR1444s* are homologs of *P. trichocarpa* and *P. euphratica MIR1444b*. zPicture (http://zpicture.dcode.org/) analysis showed that *S. purpurea* chromosome 8 and *S. suchowensis* scaffold 69 were aligned with *P. trichocarpa* chromosome 8 and *P. euphratica* scaffold 1.1 (Fig. 6e–h)^2^,^22^,^23^. However, no homologs of *P. trichocarpa* and *P. euphratica MIR1444a* were found in *S. purpurea* chromosome 8 and *S. suchowensis* scaffold 69. It was resulted from deletion of a DNA segment with length about 8 kb (Fig. 6e–h). Thus, the loss of *P. trichocarpa* and *P. euphratica MIR1444a* homologs in *S. purpurea* and *S. suchowensis* occurred after the divergence of *Populus* and *Salix* lineages.

## Discussion

Plant miR1444s are functionally significant miRNAs. They regulate copper homeostasis and stress responses through cleaving the transcripts of *PPO* genes in *P. trichocarpa*^1^,^7^,^8^,^9^. Previously, *MIR1444* genes were reported only in *P. trichocarpa*, although the mature sequences of miR1444 had been identified from *S. matsudana* and various *Populus* species^1^,^7^,^11^,^12^,^14^,^15^,^16^,^17^. In this study, we found two *MIR1444* genes in various *Populus* species and one in *Salix*. Also, we identified two *MIR1444* genes from *I. polycarpa*, a Salicaceae species most closely related to *Populus* and *Salix*^18^,^19^,^21^. This brings the number of authentic *MIR1444* precursors to 23, including 18 from *Populus*, 3 from *Salix* and 2 from *Idesia*. The 23 *MIR1444s* can be divided into two groups. *Populus* and *Idesia MIR1444b* precursors and *Salix MIR1444s* were separated from *Populus* and *Idesia MIR1444a* precursors in the phylogenetic NJ tree. It shows the divergence between *MIR1444a* and *MIR1444b* precursors in *Populus* and *Idesia* and suggests that *Salix MIR1444s* have higher level of homology with *MIR1444b* precursors than *MIR1444a* precursors in *Populus* and *Idesia*.

In *P. trichocarpa*, in addition to the precursors, divergence was also observed in mature sequences of miR1444a and miR1444b. Ptr-miR1444a and ptr-miR1444b contain two nucleotide changes (U-to-C and C-to-G) at positions close to the 3′ end. Compared with ptr-miR1444b, ptr-miR1444a started 2 nt upstream. Examination of the precursor sequences and secondary structures showed difference between *ptr-MIR1444a* and *ptr-MIR1444b*. It indicates that *DCL1*-mediated processing of primary miRNAs is probably affected by the precursor sequences and mismatch patterns between miRNA and miRNA*. The results are consistent with previous findings^51^,^52^,^53^,^54^,^55^,^56^. Although the underlying mechanism positioning the cleavage sites in *ptr-MIR1444a* and *ptr-MIR1444b* remains to be elucidated, both of the mature ptr-miR1444a and ptr-miR1444b are functional. They were validated to target a subset of *PPO* genes for cleavage in *P. trichocarpa* (Fig. 3c). In consistent with the divergence of miRNA sequences, ptr-miR1444a and ptr-miR1444b directed the cleavage of *PPO* transcripts at two positions with 2 nt distance.

*PPOs* exist widely in land plants, fungi and some bacteria^57^, although *Arabidopsis thaliana, A. lyrata* and various species of green algae do not contain *PPO* genes^43^,^58^. In plants, the number of *PPOs* varied significantly among species^43^,^59^. Through genome-wide analysis, we identified a total of 34 full-length *PPO* genes in Salicaceae plants, including 15 *PtrPPOs*, 6 *PeuPPOs*, 9 *SpuPPOs* and 4 *SsuPPOs*. Land plant *PPO* genes were originally transferred from a bacterium about 450 to 500 Ma through an ancient horizontal gene transfer event during the transition of an early common ancestor of land plants to live on land^57^. The dynamic of the *PPO* gene family reflects that *PPO* genes have undergone frequent gain and lost during plant evolution. Expansion of the *PPO* gene family in some plant species appears a consequence of gene duplication events, such as the WGD events and the tandem duplication events. The lack of *PPO* genes in *Arabidopsis* and the identification of many partial *PPO* sequences in the genomes of *P. trichocarpa, P. euphratica, S. purpurea* and *S. suchowensis* suggest that *PPO* genes may be lost through deletion and mutation during chromosome rearrangement.

**Figure 7:**
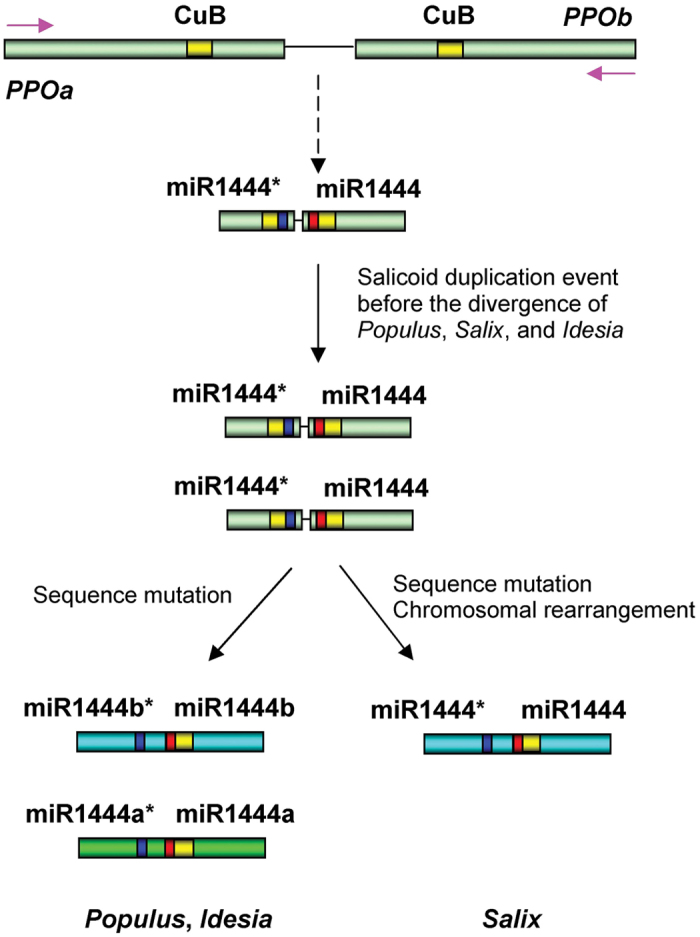
Proposed model for *MIR1444* origin and evolution. *MIR1444* genes were originated from *PPOs* through an inverted gene duplication event. It resulted in tail-to-tail orientations of complete or partial *PPO* gene sequences, which were diversified through sequence mutation to shorten and gain of bulges in the foldback structure. Continuous mutation generates *MIR1444* genes with sequences and mismatch patterns in and surrounding the miR1444:miR1444* region. *MIR1444* genes were expanded through the Salicoid genome duplication event and then further diversified through sequence mutation. A copy of the duplicated *MIR1444* genes was lost through DNA segment deletion during chromosome rearrangement in *Salix*. Pink arrows indicate transcription direction of *PPO* genes.

Based on the analysis of *Arabidopsis* and rice *MIR* loci, three possible models of *MIR* origin have been proposed^6^. In the first model, *MIR* genes were generated from inverted gene duplication events of target genes^49^,^50^. The second model termed spontaneous evolution^6^,^60^. According to this model, evolutionarily young *MIRs* were originated from high density of small-to-medium sized fold-back sequences scattered throughout the plant genome. The last model indicated the derivation of some evolutionarily young plant *MIRs* from transposable elements^6^,^61^. Through the analysis of *MIR1444* genes in *Populus, Salix* and *Idesia*, we proposed that *MIR1444* genes were originated from *PPOs* through an inverted gene duplication event (Fig. 7). This hypothesis is supported by the fact that *MIR1444* genes show extensive similarity to their *PPO* targets, and the existence of many partial *PPO* sequences in plant genomes. The inverted duplication event resulted in tail-to-tail orientations of complete or partial *PPO* gene sequences as proposed for *Arabidopsis MIR163*^49^. The tail-to-tail-orientated *PPO* sequences were diversified through sequence mutation to shorten and gain of bulges in the foldback structure. Continuous mutation generates evolutionarily young *MIR1444* genes with sequences and mismatch patterns in and surrounding the miR1444:miR1444* region to be compatible with DCL1-mediated processing^6^. It has been shown that *Salix* and *Populus* share the Salicoid duplication event estimated to be occurred around 60 to 65 Ma^2^,^22^,^23^. Through the comparative analysis of *MIR1444* genes in *Populus* and *Idesia*, we found that *Idesia* could also share the Salicoid genome duplication event and *Populus* and *Idesia MIR1444* genes were all expanded through the event (Fig. 7). After duplication, the sequence of *MIR1444* genes was further diversified through mutation as evidenced by precursors and mature miR1444 sequences in *Populus* and *Idesia* or lost through DNA segment deletion during chromosome rearrangement in *Salix* (Fig. 7).

Evolutionarily young miR1444s regulate *PPOs* in *Populus, Salix* and *Idesia*. The target site locates in a region encoding the conserved CuB domain. Duplication events yielding foldbacks from highly conserved domains are considered to be under strong negative selection, since miRNAs usually target to the regions outside of family-defining domains^49^. Thus, the origination of conserved CuB domain-targeted *MIR1444* genes could be under strong negative selection. It indicates that miR1444s are particularly important for Salicaceae plants. The generation of a miRNA targeting conserved domains is beneficial to regulate the whole gene family or the majority of family members. This type of regulation seems to be vital for a family of genes with similar and significant functions. It has been shown that cytosolic *PPOs* can trigger a stress response and cause the accumulation of anthocyanins, which eventually lead to reduce the growth rates of plants^57^. A mechanism to alleviate the effects of cytosolic PPOs is targeting them to plastids^57^. miR1444s may play a role of fine adjustment during this process. They cleave those *PPOs* without the intact chloroplast transit peptide-encoding sequence and control the level of plastid-targeted PPOs. The regulation of *PPOs* is more sophisticated in *Populus* plants, which contain sequence divergent ptc-miR1444a and ptc-miR1444b. Taken together, evolutionary force-driven origination of *MIR1444s* may be important for long-term growth and survival of Salicaceae trees in stressful environments. In view that PPOs are dynamic and flexible enzymes evolved to play a variety of specific functions in different plants^43^, miRNA-mediated and lineage-specific regulation of *PPO* genes is likely to exist in other PPO-containing plants. Consistently, a grapevine PPO has recently been found to be regulated by a novel miRNA, termed Vv-miR058^62^. Further investigating miRNAs in other plant species will help to test this hypothesis.

## Methods

### Plant materials

Seeds of *Idesia polycarpa* Maxim. var. *vestita* Diels were collected in a field nursery at Shuyang, Jiangsu Province, China. *Populus trichocarpa* (genotype Nisqually-1) plants were grown in a greenhouse for about seven months^63^. To induce shoots, stem segments of vigorously growing plants were cultivated on WPM agar media for three weeks in a controlled growth chamber under the following conditions: temperature 24–26 °C, humidity 60–70%, 16-h light/8-h darkness^7^,^64^. Shoots with 1–2 cm height were then excised and cultivated on WPM media with different zinc ion concentration for one month. Stems, leaves and roots were harvested separately, and stored immediately in liquid nitrogen.

### *Populus* and *Salix MIR1444* gene identification

*P. trichocarpa MIR1444* precursors were downloaded from miRBase^23^. Genomic loci of *MIR1444s* were identified through BLAST analysis of *ptr*-*MIR1444* precursors against the current genome assemblies of *P. trichocarpa* (v3.0), *P. euphratica* (v1.0), *S. purpurea* (v1.0) and *S. suchowensis* (v1.0) using BLASTn^2^,^22^,^23^,^29^,^65^. Transcriptome-wide identification of *MIR1444* genes was carried out through BLAST analysis of *ptr*-*MIR1444s* against the Nt and EST databases and/or RNA-seq reads from illumina and 454 runs using BLASTn^65^. The SRA accession numbers for illumina and 454 RNA-seq data are listed in Supplementary Table S1.

### Prediction of stem-Loop structures

Secondary structures were predicted by the mfold program using the default parameters^66^. In each case, only the lowest energy structure was selected as described previously^48^.

### Molecular cloning of *ipo-MIR1444a* and *ipo-MIR1444b* genes

Blast analysis of *Populus* and *Salix MIR1444* genes against RNA-seq data of *Idesia polycarpa* flower buds was carried out using BLASTn^65^. Genomic DNA was isolated from *I. polycarpa* seeds treated with distilled water for 12 h using the Plant Genomic DNA kit (Tiangen, Beijing, China). PCR amplification was performed using 100 ng genomic DNA as templates under the following conditions: pre-denaturation at 95 °C for 10 min, then 30 cycles of amplification at 94 °C for 30 s, 55 °C for 30 s and 72 °C for 1 min, followed by a final extension at 72 °C for 10 min. PCR products were gel-purified and cloned into pMD18-T vector (Takara, Shiga, Japan) for sequencing at Sangon Biotech (Shanghai, China). Primers used were listed in Supplementary Table S3.

### Small RNA library construction and high throughput sequencing

Total RNA was extracted from leaves, stems and roots of one-month old *P. trichocarpa* cultivated *in vitro* using the total RNA purification kit (LC Sciences, Houston, TX, USA). The quality and quantity of RNA were examined using gel analysis and Agilent 2100 Bioanalyzer (Agilent, Palo Alto, CA, USA). Equal amount of RNA from each tissue was mixed. A total of 1 μg high quality RNA (RNA integrity number or RIN > 8.5) was used for small RNA library construction as described^67^. Briefly, 15–30 nt small RNAs were purified from a 15% denaturing polyacrylamide gel and then ligated sequentially to 5′ and 3′ RNA/DNA chimeric oligonucleotide adaptors by T4 RNA ligase 2. RT-PCR amplification was performed. The resulting small RNA libraries were sequenced using the Genome Analyzer GA IIx (Illumina, San Diego, CA, USA) at LC Sciences (Hangzhou, China). Raw reads were firstly processed to filter out the adapter and low quality and low-copy sequences. The obtained clean small RNA sequences were mapped to the precursors of ptr-miR1444a and ptr-miR1444b using SOAP2 with no mismatch allowed^39^.

### Genome-wide identification of *PPO* genes

To identify full-length *P. trichocarpa PPO* genes, we first searched *P. trichocarpa* genome (v3.0) for the conserved pfam polyphenol oxidase middle domain (PF12142)^2^. The retrieved proteins were then analyzed for conserved domains (http://www.ncbi.nlm.nih.gov/Structure/cdd/wrpsb.cgi). Those with three conserved domains, including N-terminal targeting signal, dicopper centre and C-terminal region, were considered as full-length PtPPO proteins. Partial sequences of *P. trichocarpa PPO* genes were obtained by BLAST analysis of full-length PtPPO proteins against *P. trichocarpa* (v3.0) using tBLASTn^2^,^65^.

To identify *PPO* gene sequences in *P. euphratica, S. purpurea* and *S. suchowensis*, we carried out BLAST analysis of PtPPO proteins against the current genome assemblies of *P. euphratica* (v1.0), *S. purpurea* (v1.0) and *S. suchowensis* (v1.0) using tBLASTn^22^,^23^,^65^. An e-value cut-off of 1e–10 was applied to the homologue recognition. Gene models of *PPOs* were predicted based on the alignments between the retrieved DNA sequences and *PPOs* from other plant species using BLASTx^65^. The retrieved DNA sequences were also BLAST-analyzed against the gene models available in the databases of *P. euphratica* (v1.0), *S. purpurea* (v1.0) and *S. suchowensis* (v1.0) using BLASTx^65^.

### Bioinformatics analysis and phylogenetic tree construction

The molecular weight (MW) and theoretical isoelectric point (p*I*) were predicted using the Compute pI/MW tool on the ExPASy server (http://web.expasy.org/compute_pi/). Intron/exon structures were analyzed using GSDS 2.0^68^. Subcellular localization of PPO proteins was predicted using TargetP 1.1^46^. Conserved domains were analyzed by searching the deduced amino acid sequence of PPOs against the NCBI conserved domain (http://www.ncbi.nlm.nih.gov/Structure/cdd/wrpsb.cgi). Multiple sequence alignment was carried out using DNAMAN. Phylogenetic tree was constructed using MEGA5.0 by the neighbor-joining (NJ) method with 1000 bootstrap replicates^38^. Alignment of large genomic DNA segments was performed using zPicture (http://zpicture.dcode.org/) and Generic Synteny Browser (GBrowse-syn)^29^.

### psRNATarget analysis of miR1444 targets

Targets of ptr-miR1444a, ptr-miR1444b, peu-miR1444a, peu-miR1444b, spu-miR1444 and ssu-miR1444 were predicted using psRNATarget with the default parameters^45^. *PPO* genes identified from *P. trhichcarpa, P. euphratica, S. purpurea* and *S. suchowensis* were used as target transcript candidates. The maximum expectations of 3.0 and the target accessibility-allowed maximum energy to unpair the target site of 25.0 were applied.

### 5′ RLM-RACE validation of miR1444-directed cleavage

miR1444-directed cleavage of *PPO* genes were validated using the modified RNA ligase-mediated rapid amplification of 5′ cDNA ends method (5′ RLM-RACE) as described previously^48^. The SMARTer™ RACE cDNA Amplification kit (Clontech, Mountain View, CA, USA) was used. Total RNA was isolated from pooled tissues containing leaves and xylem of *P. trichocarpa*. The nesting and the nested PCR amplification of cleaved transcripts was performed using primers listed in Supplementary Table S3.

## Additional Information

**How to cite this article**: Wang, M. *et al*. Origin and evolution of *MIR1444* genes in Salicaceae. *Sci. Rep.* **7**, 39740; doi: 10.1038/srep39740 (2017).

**Publisher's note:** Springer Nature remains neutral with regard to jurisdictional claims in published maps and institutional affiliations.

## Supplementary Material

Supplementary Information

Supplementary Dataset 1

Supplementary Dataset 2

## Figures and Tables

**Table 1:** Sequence features of *PPO* genes in *P. trichocarpa, P. euphratica, S. purpurea* and *S. suchowensis*.

Gene	Gene model^a^	ORF^b^ (bp)	Len^c^ (aa)	MW^d^ (kDa)	p*I*^e^	Loc^f^
*PtrPPO1*	Potri.011G108300	1689	563	64.0	6.34	C
*PtrPPO2*	Potri.001G387900	1743	581	64.8	6.40	C
*PtrPPO3*	Potri.011G047300	1770	590	66.2	7.02	C
*PtrPPO4*	Potri.T062100	1635	545	60.6	6.21	C
*PtrPPO5*	Potri.001G388600	1743	581	64.6	5.91	C
*PtrPPO6*	Potri.001G388400	1743	581	64.9	6.35	C
*PtrPPO7*	Potri.001G388300	1323	441	49.5	5.13	—
*PtrPPO8*	Potri.T061900	1743	581	65.0	6.68	C
*PtrPPO9*	Potri.001G388100	1563	521	58.6	5.26	—
*PtrPPO10*	Potri.011G108200	1710	570	64.7	7.66	—
*PtrPPO11*	Potri.001G388900	1818	606	68.6	7.01	C
*PtrPPO12*	Potri.001G388800	1743	581	64.9	6.57	C
*PtrPPO13*	Potri.004G156500	1761	587	67.4	6.82	S
*PtrPPO14*	Potri.001G388200	1743	581	64.7	5.04	C
*PtrPPO15*	Potri.T062200	1743	581	64.8	6.74	C
*PeuPPO1*	CCG023279	1743	581	64.8	6.05	C
*PeuPPO2*	CCG009235	1743	581	64.9	6.71	C
*PeuPPO3*	CCG025949	1680	560	64.1	7.07	C
*PeuPPO4*	CCG012857	1770	590	66.3	7.37	C
*PeuPPO5*	CCG004943	1719	573	65.0	6.35	C
*PeuPPO6*	CCG033201	1761	587	67.5	6.60	S
*SpuPPO1*	SapurV1A.0044s0470	1779	593	66.3	7.19	C
*SpuPPO2*	SapurV1A.0737s0010	1695	565	64.6	5.86	—
*SpuPPO3*	SapurV1A.0064s0010	1746	582	65.2	6.40	C
*SpuPPO4*	SapurV1A.0064s0020	1647	549	62.3	7.38	C
*SpuPPO5*	SapurV1A.0064s0030	1746	582	65.2	6.40	C
*SpuPPO6*	SapurV1A.0064s0040	1719	573	64.7	5.91	C
*SpuPPO7*	SapurV1A.0064s0050	1785	595	66.7	5.67	—
*SpuPPO8*	SapurV1A.0064s0060	1719	573	64.5	6.05	C
*SpuPPO9*	SapurV1A.0721s0060	1722	574	65.4	6.86	S
*SsuPPO1*	willow_GLEAN_10017487	1719	573	64.7	5.93	C
*SsuPPO2*	willow_GLEAN_10010908	1779	593	66.1	7.06	C
*SsuPPO3*	willow_GLEAN_10008936	1695	565	64.5	6.12	—
*SsuPPO4*	willow_GLEAN_10000730	1713	571	64.5	7.02	C

‘S’ stands for secretory pathway. ‘C’ stands for chloroplast. ‘—’ indicates any locations other than the plastid, mitochondrion and secretory pathway.

^a^Gene models available in the databases of *P. trichocarpa* (v3.0), *P. euphratica* (v1.0), *S. purpurea* (v1.0) and *S. suchowensis* (v1.0) are shown.

^b^ORF represents open reading frame.

^c^Len represents the number of amino acid residues.

^d^MW represents molecular weight.

^e^p*I* represents theoretical isoelectric point.

^f^Loc represents the protein localization predicted by TargetP1.1.
