# Supplementary material for: Origin and evolution of MIR1444 genes in Salicaceae
Source: Sci Rep. 2017 Jan 10;7:39740. doi: 10.1038/srep39740 (PMC5223194; doi:10.1038/srep39740)
Supplement: Supplementary Information [file srep39740-s1.pdf]

Supplementary Information

Origin and evolution of *MIR1444* genes in Salicaceae

Meizhen Wang[1], Caili Li[1], Shanfa Lu[1,*]

[1] Institute of Medicinal Plant Development, Chinese Academy of Medical Sciences and Peking Union Medical College, Beijing, China

[*] Correspondence and requests for materials should be addressed to S.L. (e-mail: sflu@implad.ac.cn Tel.: 86-10-57833366 Fax: 86-10-57833366)

Table S3 Primers used in this study.

Use	Primer name	Sequence (5'-3')
Ipo-MIR1444 cloning	IpoMIR1444a-F	CCCGGTAACATTTCATTGATGGC
	IpoMIR1444a-R	GGTACTCCTGAGTGGCAAACA
	IpoMIR1444b-F	TCTTCAGGTGATGCCGATCAAG
	IpoMIR1444b-R	CGGAGGCATCTCCATGTTAGTT
ptr-miR1444 target site validation	PtrPPO2-R1	GCATGCTTGTGAGGCACATTGAT
	PtrPPO2-R2	GTTAATACAACGCTTTTTTAGCTGA
	PtrPPO2-R3	CGACGTGATTTTTCCTGCCTTGTA
	PtrPPO2-R4	GCCTTGTAAATTTTGGTGTTGCTCTAGC
	PtrPPO3-R1	CATGGCCAAAGATATTGCTAGCCA
	PtrPPO3-R2	GGTGTTGGCCTGGATTGTAGCCAA
	PtrPPO3-R3	GGTTTCTTGAATCCAGGCAATCTCT
	PtrPPO6-R1	GTCACCACAAGGGTATCATCC
	PtrPPO6-R2	CTTCATACTCAATCCCTTCAATAACC
	PtrPPO6-R3	CGACGTGATTTTTCCTGCCTTGTT
	PtrPPO9-R1	CTTCATACTCAATCCCTTCAATAACC
	PtrPPO9-R2	GCACGACGTGATTTTTCCTGCCTTGTT
	PtrPPO9-R3	GCCTTGTTAATTTTGGTGTTGCTCTAGT
	PtrPPO11-R1	GGCACTGTCGGCTTTGGTTTGTT
	PtrPPO11-R2	GGTCTAGCATTGATCCAGGGACTA
	PtrPPO11-R3	GCAACATCTTGAAACCCATATCTCAAC
	PtrPPO15-R1	GACGAGGACATCGAACTTAATTG
	PtrPPO15-R2	CTTCATACTCAATCCCTTCAATAACT
	PtrPPO15-R3	CCTGCCTTGTTAATTTTGGTGTTGC
	PtrPPO15-R4	GTTAATTTTGGTGTTGCTCTAGTTT
	NUP	CTAATACGACTCACTATAGGGC
	UPM	CTAATACGACTCACTATAGGGCAAGCAGTG GTATCAACGCAGAGT

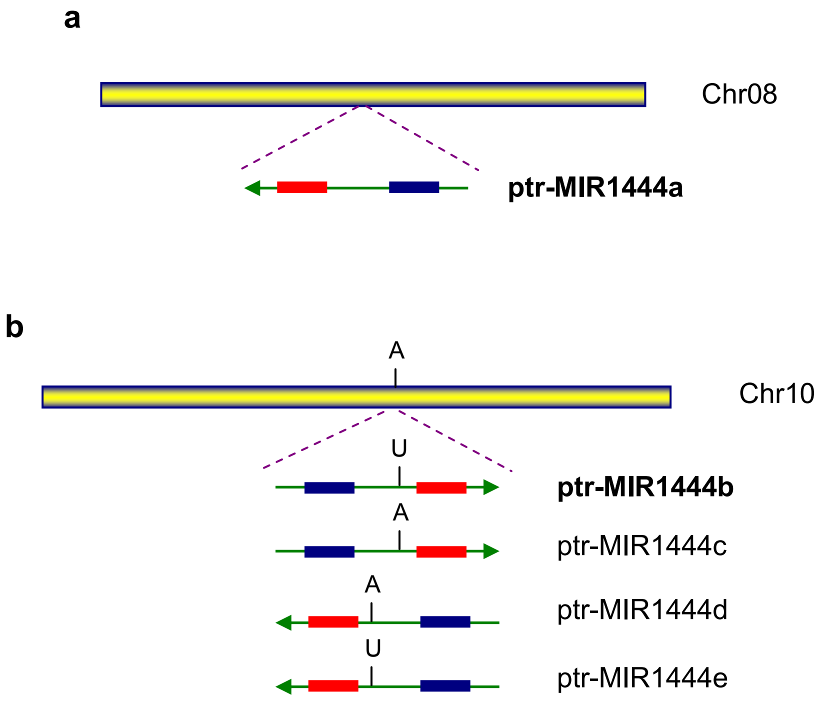

Figure S1 Blast analysis of *ptr-MIR1444* precursors against the *P. trichocarpa* genome database (v3.0). (a) *ptr-MIR1444a* locates on chromosome 8. (b) *ptr-MIR1444b* – *ptr-MIR1444e* locate on chromosome 10. Arrows indicate transcriptional direction. miRNAs are indicated in red. miRNAs* are indicated in blue. The mismatched nucleotides are shown.

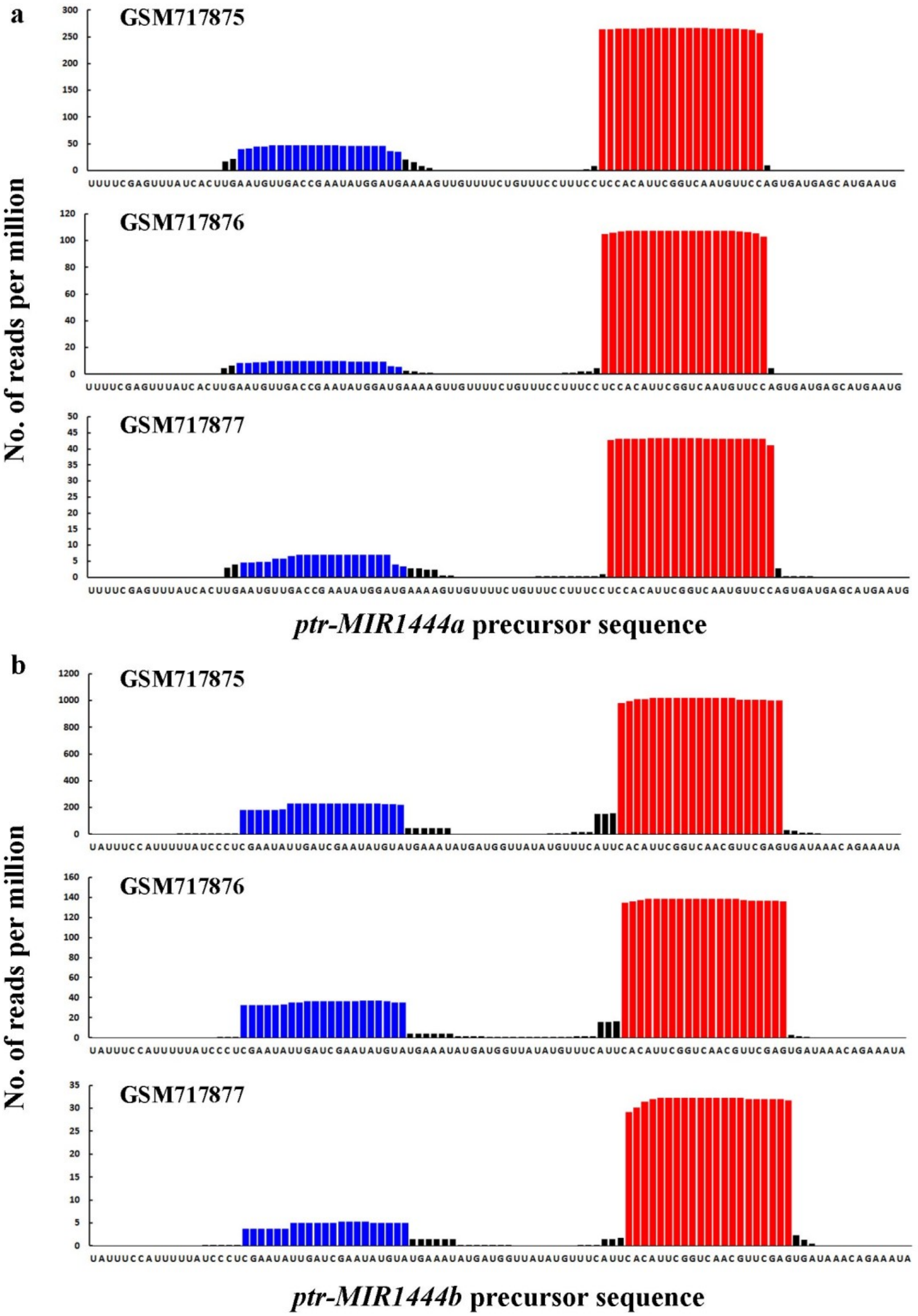

Figure S2 High throughput sequencing analysis of small RNAs from *ptr-MIR1444a* (a) and *ptr-MIR1444b* (b) precursors. Three previously reported small RNA libraries (GSM717875,

GSM717876 and GSM717877) were used (Puzey et al. 2012). The y-axis represents the normalized number of nucleotides from different reads mapping to that specific nucleotide. Red bars indicate the mature miRNA sequences, while blue bars indicate the miRNA* sequences. Black bars indicate nucleotides locating outside of the miRNA and miRNA* sequences.
